# Insights From a Scoping Review and Bibliometric Analysis of Trends and Advances in Biomarker Research: The Role of MicroRNAs in Cognitive Decline Across Neurodegenerative Diseases

**DOI:** 10.7759/cureus.98237

**Published:** 2025-12-01

**Authors:** Samuel Reyes-Long, Ernesto Roldan-Valadez, Jose-Luis Cortes-Altamirano, Denise Clavijo-Cornejo, Alfonso Alfaro-Rodriguez

**Affiliations:** 1 Division of Basic Neuroscience, Instituto Nacional de Rehabilitacion "Luis Guillermo Ibarra Ibarra", Mexico City, MEX; 2 Department of Natural Sciences, Universidad Autónoma Metropolitana - Unidad Cuajimalpa, Mexico City, MEX; 3 Division of Research, Instituto Nacional de Rehabilitacion "Luis Guillermo Ibarra Ibarra", Mexico City, MEX; 4 Department of Radiology, I.M. Sechenov First Moscow State Medical University (Sechenov University), Moscow, RUS; 5 Division of Basic Neuroscience, Instituto Nacional de Rehabilitación "Luis Guillermo Ibarra Ibarra", Mexico City, MEX; 6 Department of Research, Universidad Estatal del Valle de Ecatepec, Ecatepec de Morelos, MEX; 7 Division of Rheumatology, Instituto Nacional de Rehabilitación "Luis Guillermo Ibarra Ibarra", Mexico City, MEX

**Keywords:** bibliometric analysis, citation classics, cognitive impairment, dementia, micrornas, scoping review

## Abstract

MicroRNAs (miRNAs) are small non-coding RNA molecules that negatively regulate gene expression and are implicated in the pathogenesis of neurodegenerative diseases, such as Alzheimer's disease (AD) and Parkinson's disease (PD). This study aims to provide a bibliometric analysis of the most influential research on miRNAs related to cognitive decline in AD and PD. Searches were run in Scopus on July 23, 2024 (verification July 29, 2024) for records 2014-2021; journal indicators were retrieved from Clarivate Journal Citation Reports (JCR) at the time of extraction. Selection was based on total citations; citations per year were calculated for interpretation. The search initially identified 7,722 articles related to miRNAs and cognitive decline in AD and PD. After applying predefined inclusion and exclusion criteria, the top 100 most-cited articles, published between 2015 and 2021, were selected for detailed analysis. Citation metrics and bibliometric data were extracted and analyzed to determine trends, influential journals, key authors, and leading institutions. The analysis revealed a peak in publications in 2014, with 28 articles contributing to a total of 5,339 citations. Among the top 100 articles, 60 were classified as Citation Classics (≥100 citations), and 40 were identified as Hyperclassics (≥250 citations). The leading journals were Molecular Neurobiology, Progress in Neurobiology, and PLoS ONE, collectively accounting for 2,944 citations (14.7% of the total). Harvard Medical School emerged as the top contributing institution, with four highly cited articles totaling 683 citations (3.3% of the total). Geographically, the United States led the research landscape with 31 articles, representing 32.1% (6,593 citations) of the total citations. This bibliometric analysis highlights the significant role of miRNAs in cognitive decline related to AD and PD. Review articles comprised 65% of the Hyperclassics, emphasizing their impact in consolidating and advancing knowledge. The findings underscore the importance of continuous innovation, interdisciplinary collaboration, and global research efforts in the development of miRNAs as biomarkers for early diagnosis and monitoring of cognitive decline in neurodegenerative diseases.

## Introduction and background

MicroRNAs (miRNAs or miRs) are small, non-coding RNAs that post-transcriptionally regulate gene expression by promoting messenger RNA (mRNA) degradation or inhibiting translation [1]. They participate in fundamental cellular processes - including cell-to-cell communication, proliferation, and apoptosis - thereby influencing normal physiology and disease pathways [2]. miRNAs are attractive for clinical translation because they are detectable in most biofluids and remain relatively stable during routine handling; together with their disease-specific expression patterns, these features support their promise as biomarkers and therapeutic targets across conditions [3,4]. In the context of cognition, miRNAs have emerged as potential early-stage indicators of neurodegenerative disease and may inform trajectories of decline [5,6]. This review extends prior syntheses by coupling citation influence with science-mapping and a scoping-review lens, allowing us to situate highly cited findings within translational pathways (e.g., early detection, risk stratification, treatment monitoring) rather than reporting citation counts alone.

Cognitive impairment (CI) - a decline in memory, attention, or other cognitive domains - is often an early manifestation of central nervous system (CNS) injury [7]. The rising prevalence of neurodegenerative disorders reflects aging populations and interacting genetic and environmental risks [8]. Alzheimer’s disease (AD) and Parkinson’s disease (PD) are the most common neurodegenerative causes of CI [9]. In AD, CI ranges from mild cognitive impairment (MCI) to dementia, with variable progression and incompletely understood mechanisms [10]. In PD, attentional, executive, visuospatial, and memory deficits may appear early and typically worsen over time [9].

From a clinical perspective, distinguishing age-related change from prodromal neurodegeneration is challenging. While CSF assays and amyloid/tau Positron Emission Tomography (PET) can assist, they are invasive or costly and not universally accessible. By contrast, circulating miRNAs - measured in plasma, serum, or CSF and often protected within extracellular vesicles - are analytically stable and amenable to serial sampling for early detection, risk stratification, and longitudinal monitoring of cognitive decline [3-6]. Mechanistically, miRNAs intersect with key pathological pathways relevant to cognition, including amyloid-β aggregation, tau phosphorylation, and synaptic plasticity.

The scientific output on miRNAs has expanded rapidly across medical fields [11-13]. Prior syntheses have underscored the role of non-coding RNAs in AD research [14]. However, a focused map of the most influential literature on miRNA biomarkers related specifically to cognitive decline across AD and PD - linking themes, collaboration networks, and translational directions - has been limited. The present bibliometric-scoping review addresses this gap by analyzing the top-cited corpus to highlight research influence and emerging clinical relevance. This approach is intended to guide hypothesis generation, inform study design, and support clinically oriented readers entering the field [15].

Recent syntheses - including a systematic review/meta-analysis of biofluid miRNAs in PD [16] and extracellular vesicle (EV)-miRNA overviews in AD - have summarized diagnostic performance and mechanistic links [17,18]. Our study differs by analyzing the top-cited corpus to map influential themes, collaboration patterns, and journal impact - information that complements traditional systematic reviews and is meant to guide hypothesis generation and clinical translation.

Recent translational studies reinforce this rationale: blood-derived miRNAs correlate with specific cognitive domains in population samples, supporting their use as early, subclinical indicators of decline [19]. In AD cohorts, miR-129-5p relates to pathology and longitudinal cognition, and EV-associated miRNA panels show diagnostic utility across the A/T/N framework [17,18,20]. Complementarily, machine-learning models using miRNA features have achieved high diagnostic performance, underscoring their feasibility for scalable screening [21]. For PD, recent syntheses and causal-inference analyses highlight diagnostic potential and nominate miRNAs implicated in PD dementia, extending clinical relevance beyond AD [16,22].

Objective of the review

To provide a comprehensive bibliometric analysis of the most-cited literature on miRNAs and cognitive decline in AD and PD - identifying influential studies, research themes, and collaboration networks - and to emphasize the translational potential of miRNA biomarkers for early diagnosis, disease monitoring, and therapeutic response in neurodegenerative disease. Focusing on the top-100 most-cited articles captures influence within the field; it does not imply a higher level of evidence strength, which must be established through prospective validation.

Although our corpus centers on AD/PD, the vascular pathways that drive cognitive decline - endothelial dysfunction, blood-brain barrier disruption, microvascular injury, and sterile inflammation - overlap with miRNA biology captured in this review. Consequently, the themes we map (circulating/EV miRNAs, inflammatory and neurovascular signaling) are directly relevant to post-stroke cognitive impairment and vascular dementia.

## Review

Materials and methods

Study Design

This is a bibliometric-scoping review of the top-cited literature on miRNAs related to cognitive impairment/decline in AD and PD, reported following Preferred Reporting Items for Systematic Reviews and Meta-Analyses (PRISMA)-Scoping Review guidelines [23]. The aim was to map influential themes and translational directions rather than to pool clinical effects.

Eligibility Criteria

We included English-language human studies (original articles and reviews) that reported or analyzed miRNA measurements related to cognition in AD or PD (biofluids: plasma/serum/CSF; or tissue when explicitly linked to cognitive outcomes). We excluded conference papers, books, notes, editorials, short surveys, letters, and unrelated indications. Records were ranked by total citations to identify the top-100 most-cited studies; citation rank reflects research influence, not evidence hierarchy.

Information Sources and Search Strategy

A primary search was conducted in Scopus (https://www.scopus.com) on 23 July 2024 using TITLE-ABS-KEY field tags and predefined concept groups for miRNAs, cognition, and AD/PD. We selected Scopus because of its broad biomedical coverage, robust citation/export utilities, and standardized author/affiliation metadata; journal indicators were verified in Clarivate Journal Citation Reports (JCR). Although database-dependent discrepancies can occur, Scopus provides consistent, reproducible citation data for ranking in bibliometric studies. Filters applied were publication years 2014-2021, document type (Article OR Review), and language (English). The concept groups are summarized in Table 1, which also reproduces the full Boolean string (verbatim) and the exact filters.

**Table 1:** Search Strategy and Categorization of Topics Related to miRNAs, Cognitive Decline, AD, and PD. This table describes the keywords and search terms used to identify relevant studies on miRNAs associated with cognitive decline in Alzheimer's and Parkinson's diseases. Scopus Boolean query (verbatim) and filters used for retrieval (field tags, document types, language, and year window 2014–2021). Abbreviations: AD, Alzheimer’s disease; PD, Parkinson’s disease; miRNA, microRNA.

Primary Focus and Context	Explanation	Scopus Search Algorithm	Boolean Connector
Proposed biomarker molecule.	Give us information related to microRNA expression profiles, it covers known abbreviations.	("microRNA" OR "microRNAs" OR "miRNA" OR "miRs")	AND
A symptom prevalent in neurodegenerative diseases.	Covers the terms employed for the combination and each component of cognitive manifestations.	("cognitive impairment" OR "cognitive decline" OR "memory" OR "attention")	AND
Specific neurodegenerative disease.	Specifies the pathology in which the miRNA expression and the cognitive symptoms are evaluated.	("Alzheimer's disease" OR "Alzheimer")	OR
Specific neurodegenerative disease.	Specifies the pathology in which the miRNA expression and the cognitive symptoms are evaluated.	("Parkinson's disease" OR "Parkinson")	OR
Exclusion Keywords	Keywords to exclude other pathologies which were not on the focus of the review or could change miRNA expression by themselves.	("cancer" OR "oncology" OR "cardiovascular" OR "diabetes" OR "inflammation")	NOT
Complete Scopus Search Algorithm			
(TITLE-ABS-KEY ( ( "cognitive impairment" OR "cognitive decline" OR "memory" OR "attention" ) ) OR TITLE-ABS-KEY ( ( "Alzheimer's disease" OR "Alzheimer" ) ) OR TITLE-ABS-KEY ( ( "Parkinson's disease" OR "Parkinson" ) ) AND TITLE-ABS-KEY ( ( "microRNA" OR "microRNAs" OR "miRNA" OR "miRs" ) ) AND NOT TITLE-ABS-KEY ( ( "cancer" OR "oncology" OR "cardiovascular" OR "diabetes" OR "inflammation" ) ) )			

Journal impact indicators - Journal Impact Factor (JIF) and quartiles (Q1-Q4) - were retrieved from JCR (https://jcr.clarivate.com) at the time of extraction. A verification cross-search using the same terms was performed on 29 July 2024 with no changes in the ranked top-100 set.

Screening Process

**Figure 1:**
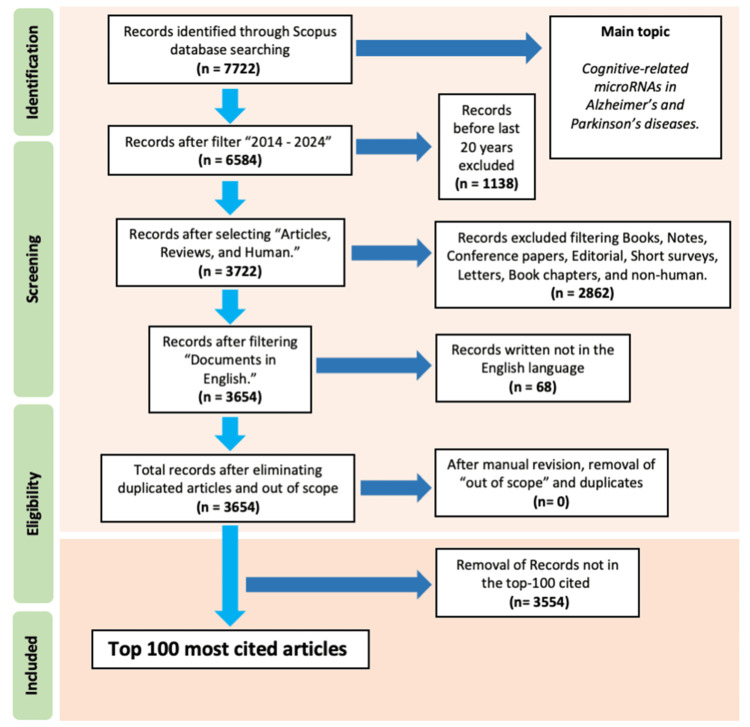
Preferred Reporting Items for Systematic Reviews and Meta-Analyses (PRISMA) Flow of Study Selection for the Top 100 Most-Cited Articles on Cognitive-Related miRNAs in AD and PD. PRISMA-style diagram of the search and selection process. Identification: Records found in Scopus (n = 7,722). Screening: Time filter 2014–2024 applied (excluded n = 1,138); article type and population filters retained Articles/Reviews and Human studies (remaining n = 3,722; excluded Books, Notes, Conference papers, Editorials, Short surveys, Letters, Book chapters, and non-human, n = 2,862); English-language restriction applied (remaining n = 3,654; excluded non-English n = 68). Eligibility: Manual check for out-of-scope items and duplicates (excluded n = 0). Included: Top 100 most-cited records retained (excluded not in top-100 n = 3,554). Abbreviations: AD, Alzheimer’s disease; PD, Parkinson’s disease; miRNA, microRNA.

After export, duplicates were removed in Microsoft Excel (Microsoft Corporation, Redmond, WA, USA). Two reviewers independently screened titles/abstracts and full texts against prespecified criteria; disagreements were resolved by consensus, with a third reviewer adjudicating when required (PRISMA-ScR). The selection process is summarized in Figure 1.

Data Extraction

In duplicate, we captured study type, disease (AD/PD), specimen/source (plasma, serum, CSF, tissue), cognitive endpoints, assay platform and normalization approach, publication year, and citation counts. For journals we recorded JIF, CiteScore, SCImago Journal Rank (SJR), Source Normalized Impact per Paper (SNIP), and quartile. Discrepancies were reconciled before analysis.

Citation Ranking and Definitions

Following the classic framework introduced by Garfield [24-26], we defined Citation Classics as articles with ≥100 citations and Hyperclassics as ≥250 citations. Derived variables included citations per year = total citations ÷ (2024 − publication year + 1) and years active = (2024 − publication year + 1).

Bibliometric Analysis Techniques

We combined performance analysis and science mapping [24,25]. Performance indicators included total publications (TP), number of active years (NAY), articles per active year (APY), number of contributing authors (NCA), and authorship pattern (sole-authored vs co-authored). Science mapping characterized the intellectual and collaborative structure using co-citation, keyword co-occurrence, and co-authorship networks to identify clusters and thematic linkages related to miRNAs and cognitive decline.

Statistical Analysis

Analyses were descriptive and exploratory. Continuous variables were compared between citation classes using the Mann-Whitney U/W test with rank-biserial effect size and 95% CI; categorical variables used chi-square (χ²) with Cramér’s V and 95% CI. Associations among journal metrics were examined with Spearman’s ρ (95% CI via Fisher’s z). Journal indicators (JIF, CiteScore, SJR, SNIP) are summarized by median, quartiles, and interquartile range (IQR). Tests were two-sided with α = 0.05; p-values are interpreted alongside effect sizes and confidence intervals [27,28]. This review did not perform meta-analysis or meta-regression. Total citations determined ranking; citations per year were also computed and reported to contextualize time-at-risk effects on citation accrual.

Software

Data handling used Microsoft Excel v16.67; statistical analyses were performed in R v4.4.1 (R Foundation for Statistical Computing, Vienna, Austria; www.r-project.org) with bibliometrix/biblioshiny for performance analysis [29]. Science-mapping visualizations were generated in VOSviewer v1.6.20 (Leiden University, Leiden, The Netherlands) following published guidance [30].

Data Availability

The curated bibliometric dataset (CSV) and R scripts are available from the corresponding author upon reasonable request.

Results

Retrieval and Selection of Articles

The Scopus search retrieved 7,722 records related to miRNAs and cognitive impairment in AD and PD. After applying the predefined criteria and removing duplicates, the top 100 most-cited articles were retained for analysis and ordered by total citations (Table 2). The selection pathway is depicted in the PRISMA-ScR flowchart (Figure 1).

**Table 2:** Top 100 most cited papers on cognitive related microRNAs in AD and PD. This table shows the complete list of included articles in the review, including bibliometric information such as the publication year, times cited, and average citation per year. Abbreviations: AD, Alzheimer’s disease; PD, Parkinson’s disease.

Ranking	Title	1st Author	Journal	Publication Year	Times Cited	Average Citation per Year
1	Therapeutic miRNA and siRNA: moving from bench to clinic as next generation medicine [31]	Chakraborty C.	Molecular Therapy Nucleic Acids	2017	601	75
2	Mesenchymal stem cell-derived extracellular vesicles: novel frontiers in regenerative medicine [32]	Keshtkar S.	Stem Cell Research and Therapy	2018	578	83
3	Parkinson's disease: a review [33]	Beitz J.M.	Frontiers in Bioscience - Scholar	2014	495	45
4	Post-stroke dementia - a comprehensive review [34]	Mijajlović M.D.	BMC Medicine	2017	440	55
5	Altered microRNA profiles in cerebrospinal fluid exosome in Parkinson disease and Alzheimer disease [35]	Gui Y.	Oncotarget	2015	412	41
6	Stress-induced perinatal and transgenerational epigenetic programming of brain development and mental health [36]	Babenko O.	Neuroscience and Biobehavioral Reviews	2015	402	40
7	Exosomes from human umbilical cord blood accelerate cutaneous wound healing through miR-21-3p-mediated promotion of angiogenesis and fibroblast function [37]	Hu Y.	Theranostics	2018	389	56
8	The β-secretase BACE1 in Alzheimer's disease [38]	Hampel H.	Biological Psychiatry	2021	374	94
9	The complexity of miRNA-mediated repression [1]	Wilczynska A.	Cell Death and Differentiation	2015	362	36
10	Dendritic spines: the locus of structural and functional plasticity [39]	Sala C.	Physiological Reviews	2014	361	33
11	Parkinson's disease: biomarkers, treatment, and risk factors [40]	Emamzadeh F.N.	Frontiers in Neuroscience	2018	348	50
12	Common features of microRNA target prediction tools [41]	Peterson S.M.	Frontiers in Genetics	2014	340	31
13	An update on diagnostic and prognostic biomarkers for traumatic brain injury [42]	Wang K.K.	Expert Review of Molecular Diagnostics	2018	331	47
14	Plasma exosomal miRNAs in persons with and without Alzheimer disease: altered expression and prospects for biomarkers [43]	Lugli G.	PLoS ONE	2015	308	31
15	Prognostic serum miRNA biomarkers associated with Alzheimer's disease shows concordance with neuropsychological and neuroimaging assessment [44]	Cheng L.	Molecular Psychiatry	2015	301	30
16	Reversing a model of Parkinson’s disease with in situ converted nigral neurons [45]	Qian H.	Nature	2020	294	59
17	Amyloid beta and phosphorylated tau-induced defective autophagy and mitophagy in Alzheimer’s disease [46]	Reddy P.H.	Cells	2019	290	48
18	Extracellular vesicles in neurodegenerative disease-pathogenesis to biomarkers [47]	Thompson A.G.	Nature Reviews Neurology	2016	289	32
19	The epigenetics of aging and neurodegeneration [48]	Lardenoije R.	Progress in Neurobiology	2015	284	28
20	microRNA dysregulation in neurodegenerative diseases: a systematic review [49]	Juźwik C.A.	Progress in Neurobiology	2019	277	46
21	Exosomes: mediators of neurodegeneration, neuroprotection and therapeutics [50]	Kalani A.	Molecular Neurobiology	2014	265	24
22	BNPMDA: Bipartite network projection for MiRNA–Disease association prediction [51]	Chen X.	Bioinformatics	2018	264	38
23	Deficiency in the ubiquitin conjugating enzyme UBE2A in Alzheimer’s disease (AD) is linked to deficits in a natural circular miRNA-7 sponge (circRNA; ciRS-7) [52]	Zhao Y.	Genes	2016	255	28
24	Systematic review of miRNA as biomarkers in Alzheimer’s disease [53]	Swarbrick S.	Molecular Neurobiology	2019	249	42
25	Data descriptor: a multi-omic atlas of the human frontal cortex for aging and Alzheimer’s disease research [54]	De Jager P.L.	Scientific Data	2018	247	35
26	The future of blood-based biomarkers for Alzheimer's disease [55]	Henriksen K.	Alzheimer's and Dementia	2014	247	22
27	MicroRNA-125b induces tau hyperphosphorylation and cognitive deficits in Alzheimer's disease [56]	Banzhaf-Strathmann J.	EMBO Journal	2014	245	22
28	Mesenchymal stem cell derived-exosomes: a modern approach in translational medicine [57]	Nikfarjam S.	Journal of Translational Medicine	2020	240	48
29	Profiles of extracellular miRNA in cerebrospinal fluid and serum from patients with Alzheimer's and Parkinson's diseases correlate with disease status and features of pathology [58]	Burgos K.	PLoS ONE	2014	240	22
30	Astrogliosis: an integral player in the pathogenesis of Alzheimer's disease [59]	Osborn L.M.	Progress in Neurobiology	2016	230	26
31	Gene therapy for neurological disorders: progress and prospects [60]	Deverman B.E.	Nature Reviews Drug Discovery	2018	230	33
32	Crosstalk between astrocytes and microglia: an overview [61]	Matejuk A.	Frontiers in Immunology	2020	227	45
33	Are circulating microRNAs peripheral biomarkers for Alzheimer's disease? [62]	Kumar S.	Biochimica et Biophysica Acta - Molecular Basis of Disease	2016	224	25
34	An atlas of cortical circular RNA expression in Alzheimer disease brains demonstrates clinical and pathological associations [63]	Dube U.	Nature Neuroscience	2019	224	37
35	Focus on extracellular vesicles: physiological role and signalling properties of extracellular membrane vesicles [64]	Iraci N.	International Journal of Molecular Sciences	2016	217	24
36	MicroRNAs in plasma and cerebrospinal fluid as potential markers for Alzheimer's disease [65]	Kiko T.	Journal of Alzheimer's Disease	2014	213	19
37	lncRNA/MicroRNA interactions in the vasculature [66]	Ballantyne M.D.	Clinical Pharmacology and Therapeutics	2016	213	24
38	Antisense oligonucleotides: translation from mouse models to human neurodegenerative diseases [67]	Schoch K.M.	Neuron	2017	212	27
39	Emerging roles of extracellular vesicles in the nervous system [68]	Rajendran L.	Journal of Neuroscience	2014	206	19
40	MicroRNAs in Alzheimer's disease: differential expression in hippocampus and cell-free cerebrospinal fluid [69]	Müller M.	Neurobiology of Aging	2014	204	19
41	Exosomes as a nanodelivery system: a key to the future of neuromedicine? [70]	Aryani A.	Molecular Neurobiology	2016	199	22
42	Circulating miR-125b as a biomarker of Alzheimer's disease [71]	Tan L.	Journal of the Neurological Sciences	2014	187	17
43	The serum exosome derived microRNA−135A, −193b, and −384 were potential Alzheimer’s disease biomarkers [72]	Yang T.T.	Biomedical and Environmental Sciences	2018	185	26
44	MicroRNA profiling of CSF reveals potential biomarkers to detect Alzheimer's disease [73]	Denk J.	PLoS ONE	2015	184	18
45	Genome-wide serum microRNA expression profiling identifies serum biomarkers for Alzheimer's disease [74]	Tan L.	Journal of Alzheimer's Disease	2014	183	17
46	Focus on extracellular vesicles: exosomes and their role in protein trafficking and biomarker potential in Alzheimer’s and Parkinson’s disease [75]	Vella L.J.	International Journal of Molecular Sciences	2016	181	20
47	An integrated multi-omics approach identifies epigenetic alterations associated with Alzheimer’s disease [76]	Nativio R.	Nature Genetics	2020	181	36
48	Circulating miRNAs as potential biomarkers in Alzheimer’s disease [77]	Galimberti D.	Journal of Alzheimer's Disease	2014	175	16
49	MiR-132/212 deficiency impairs tau metabolism and promotes pathological aggregation in vivo [78]	Smith P.Y.	Human Molecular Genetics	2015	175	18
50	Blood-based molecular biomarkers for Alzheimer's disease [79]	Zetterberg H.	Molecular Brain	2019	172	29
51	MiR-124 regulates apoptosis and autophagy process in MPTP model of Parkinson's disease by targeting to bim [80]	Wang H.	Brain Pathology	2016	172	19
52	Circulating microRNAs as potential biomarkers for psychiatric and neurodegenerative disorders [81]	van den Berg M.M.J.	Progress in Neurobiology	2020	168	34
53	MicroRNA-193b is a regulator of amyloid precursor protein in the blood and cerebrospinal fluid derived exosomal microRNA-193b is a biomarker of Alzheimer's disease [82]	Liu C.-G.	Molecular Medicine Reports	2014	166	15
54	Serum MicroRNA profiles serve as novel biomarkers for the diagnosis of Alzheimer's disease [83]	Dong H.	Disease Markers	2015	165	17
55	Dysregulation of microRNA-219 promotes neurodegeneration through post-transcriptional regulation of tau [84]	Santa-Maria I.	Journal of Clinical Investigation	2015	162	16
56	Regulatory Role of Circular RNAs and Neurological Disorders [85]	Floris G.	Molecular Neurobiology	2017	162	20
57	Circulating miRNAs as biomarkers for neurodegenerative disorders [86]	Grasso M.	Molecules	2014	160	15
58	A novel microRNA-124/PTPN1 signal pathway mediates synaptic and memory deficits in Alzheimer’s disease	Wang X.	Biological Psychiatry	2018	158	23
59	MicroRNA-339-5p down-regulates protein expression of β-site amyloid precursor protein-cleaving enzyme 1 (BACE1) in human primary brain cultures and is reduced in brain tissue specimens of Alzheimer disease subjects [87]	Long J.M.	Journal of Biological Chemistry	2014	158	14
60	Neurodevelopmental and neuropsychiatric disorders represent an interconnected molecular system [88]	Cristino A.S.	Molecular Psychiatry	2014	157	14
61	MicroRNA biomarkers of Parkinson's disease in serum exosome-like microvesicles [89]	Cao X.-Y.	Neuroscience Letters	2017	154	19
62	SNPs in microRNA target sites and their potential role in human disease [90]	Moszyńska A.	Open Biology	2017	153	19
63	Functional roles and networks of non-coding RNAs in the pathogenesis of neurodegenerative diseases [91]	Wu Y.-Y.	Journal of Biomedical Science	2020	152	30
64	MicroRNA-124 loaded nanoparticles enhance brain repair in Parkinson's disease [92]	Saraiva C.	Journal of Controlled Release	2016	140	16
65	Inhibition of miR-34b and miR-34c enhances α-synuclein expression in Parkinson's disease [93]	Kabaria S.	FEBS Letters	2015	139	14
66	Identification of circulating microRNAs for the differential diagnosis of Parkinson's disease and multiple system atrophy [94]	Vallelunga A.	Frontiers in Cellular Neuroscience	2014	139	13
67	Expression of microRNA-34a in Alzheimer's disease brain targets genes linked to synaptic plasticity, energy metabolism, and resting state network activity [95]	Sarkar S.	Brain Research	2016	138	15
68	MicroRNA-seq data analysis pipeline to identify blood biomarkers for Alzheimer’s disease from public data [96]	Satoh J.-I.	Biomarker Insights	2015	135	14
69	Exosomal miRNAs in central nervous system diseases: biomarkers, pathological mediators, protective factors and therapeutic agents [97]	Xia X.	Progress in Neurobiology	2019	134	22
70	Identification of a panel of five serum miRNAs as a biomarker for Parkinson's disease [98]	Ding H.	Parkinsonism and Related Disorders	2016	133	15
71	MIR-1, miR-10b, miR-155, and miR-191 are novel regulators of BDNF [99]	Varendi K.	Cellular and Molecular Life Sciences	2014	130	12
72	MiR128 up-regulation correlates with impaired amyloid β(1-42) degradation in monocytes from patients with sporadic Alzheimer's disease [100]	Tiribuzi R.	Neurobiology of Aging	2014	128	12
73	The role of extracellular Tau in the spreading of neurofibrillary pathology [101]	Medina M.	Frontiers in Cellular Neuroscience	2014	126	11
74	miRNA expression profiles in cerebrospinal fluid and blood of patients with Alzheimer's disease and other types of dementia - an exploratory study [102]	Sørensen S.S.	Translational Neurodegeneration	2016	126	14
75	Serum microRNA miR-501-3p as a potential biomarker related to the progression of Alzheimer's disease [103]	Hara N.	Acta neuropathologica communications	2017	125	16
76	MiR-126 contributes to Parkinson's disease by dysregulating the insulin-like growth factor/phosphoinositide 3-kinase signaling [104]	Kim W.	Neurobiology of Aging	2014	125	11
77	miR-212 and miR-132 are downregulated in neurally derived plasma exosomes of Alzheimer’s patients [105]	Cha D.J.	Frontiers in Neuroscience	2019	125	21
78	Increased microRNA-34c abundance in Alzheimer's disease circulating blood plasma [106]	Bhatnagar S.	Frontiers in Molecular Neuroscience	2014	125	11
79	microRNA profiles in Parkinson's disease prefrontal cortex [107]	Hoss A.G.	Frontiers in Aging Neuroscience	2016	125	14
80	MicroRNA-455-3p as a potential peripheral biomarker for Alzheimer's disease [108]	Kumar S.	Human Molecular Genetics	2017	124	16
81	MicroRNAs in Alzheimer’s disease [109]	Wang M.	Frontiers in Genetics	2019	123	21
82	MicroRNA-29a is a candidate biomarker for Alzheimer’s disease in cell-free cerebrospinal fluid [110]	Müller M.	Molecular Neurobiology	2016	122	14
83	MicroRNA expression levels are altered in the cerebrospinal fluid of patients with young-onset Alzheimer’s disease [111]	McKeever P.M.	Molecular Neurobiology	2018	121	17
84	The Aerobic and Cognitive Exercise Study (ACES) for community-dwelling older adults with or at-risk for mild cognitive impairment (MCI): neuropsychological, neurobiological and neuroimaging outcomes of a randomized clinical trial [112]	Anderson-Hanley C.	Frontiers in Aging Neuroscience	2018	121	17
85	MicroRNA profile in patients with Alzheimer's disease: analysis of miR-9-5p and miR-598 in raw and exosome enriched cerebrospinal fluid samples [113]	Riancho J.	Journal of Alzheimer's Disease	2017	120	15
86	Salivary biomarkers for the diagnosis and monitoring of neurological diseases [114]	Farah R.	Biomedical Journal	2018	117	17
87	Na, K-ATPase α3 is a death target of Alzheimer patient amyloid-β assembly [115]	Ohnishi T.	Proceedings of the National Academy of Sciences of the United States of America	2015	117	12
88	Brain-specific knockdown of miR-29 results in neuronal cell death and ataxia in mice [116]	Roshan R.	RNA	2014	116	11
89	MicroRNAs and synaptic plasticity-a mutual relationship [117]	Aksoy-Aksel A.	Philosophical Transactions of the Royal Society B: Biological Sciences	2014	116	11
90	Circular HDAC9/microRNA-138/Sirtuin-1 pathway mediates synaptic and amyloid precursor protein processing deficits in Alzheimer’s disease [118]	Lu Y.	Neuroscience Bulletin	2019	115	19
91	miR-7 and miR-153 protect neurons against MPP+-induced cell death via upregulation of mTOR pathway [119]	Fragkouli A.	Frontiers in Cellular Neuroscience	2014	114	10
92	Plasma miR-34a-5p and miR-545-3p as early biomarkers of Alzheimer’s disease: potential and limitations [120]	Cosín-Tomás M.	Molecular Neurobiology	2017	114	14
93	miR-132 loss de-represses ITPKB and aggravates amyloid and TAU pathology in Alzheimer's brain [121]	Salta E.	EMBO Molecular Medicine	2016	113	13
94	Profile of 6 microRNA in blood plasma distinguish early stage Alzheimer's disease patients from non-demented subjects [122]	Nagaraj S.	Oncotarget	2017	112	14
95	Identification of blood serum micro-RNAs associated with idiopathic and LRRK2 Parkinson's disease [123]	Botta-Orfila T.	Journal of Neuroscience Research	2014	111	10
96	miRNAs in synapse development and synaptic plasticity [124]	Hu Z.	Current Opinion in Neurobiology	2017	109	14
97	Emergence of exosomal miRNAs as a diagnostic biomarker for Alzheimer's disease [125]	Van Giau V.	Journal of the Neurological Sciences	2016	109	12
98	Novel upregulation of amyloid-β precursor protein (APP) by microRNA-346 via targeting of APP mRNA 5′-untranslated region: implications in Alzheimer’s disease [126]	Long J.M.	Molecular Psychiatry	2019	108	18
99	Exploratory study on microRNA profiles from plasma-derived extracellular vesicles in Alzheimer's disease and dementia with Lewy bodies [127]	Gámez-Valero A.	Translational Neurodegeneration	2019	107	18
100	MicroRNAs in brain development and function: a matter of flexibility and stability [128]	Follert P.	Frontiers in Molecular Neuroscience	2014	107	10

Performance Analysis

**Figure 2:**
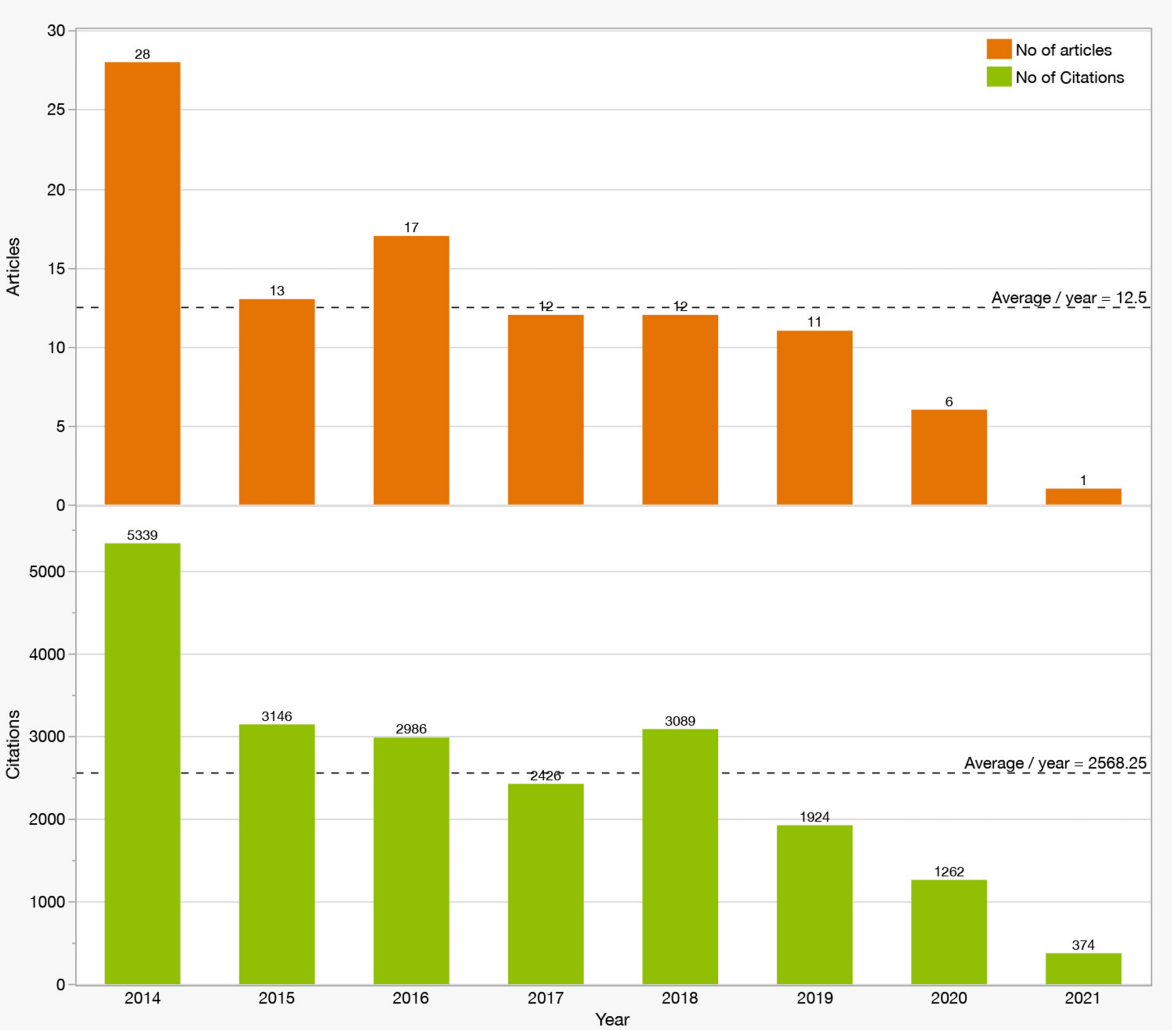
Yearly Publication Counts and Citation Accrual for the Top 100 miRNA Articles in AD/PD (2014–2021). (A) Number of articles per publication year among the top 100 most-cited studies on cognitive-related miRNAs in Alzheimer’s and Parkinson’s disease (2014–2021). The dashed line denotes the overall mean (12.5 articles/year). (B) Total citations accrued by those articles, grouped by publication year, at the time of data extraction. The dashed line denotes the overall mean (2,568.25 citations/year). Bars are labeled with counts. Axes: x-axis = publication year; y-axes = number of articles (A) and total citations (B). Abbreviations: AD, Alzheimer’s disease; PD, Parkinson’s disease; miRNA, microRNA.

Yearly number of articles and citations: Across 2014-2021, publication activity averaged 12.5 articles/year. The peak occurred in 2014 with 28 publications and 5,339 citations, reflecting a period of intensive output and influence (Figure 2A-2B). By design, the review window ended in 2021; therefore, no publications from 2022-2024 were eligible.

Citation Classics and Hyperclassics

Of the 100 articles, 60 qualified as Citation Classics (≥100 citations) and 40 as Hyperclassics (≥250 citations). As summarized in Table 3, Hyperclassics accrued more total citations and more citations per year than Classics (Mann-Whitney U, both p<0.0001), with no differences in years active or number of authors (p=0.452 and p=0.648, respectively).

**Table 3:** Continuous Performance Metrics by Citation Class. Values are mean (SD). Comparisons between Hyperclassic (≥250 citations) and Classic (≥100 citations) articles used the Mann–Whitney U test. Two-tailed; p<0.05 considered significant.

Performance variable	All articles	Hyperclassic (N=40)	Classic (N=60)	p-value
Citations	205.5 (102.9)	303.2 (97.7)	140.3 (26.0)	<0.0001
Average citations per year	25.5 (15.8)	38.5 (17.2)	16.8 (5.5)	<0.0001
Years active	8.7 (2.0)	8.5 (2.1)	8.9 (1.9)	0.452
No. of authors	8.4 (11.3)	9.8 (16.5)	7.5 (5.7)	0.648

**Figure 3:**
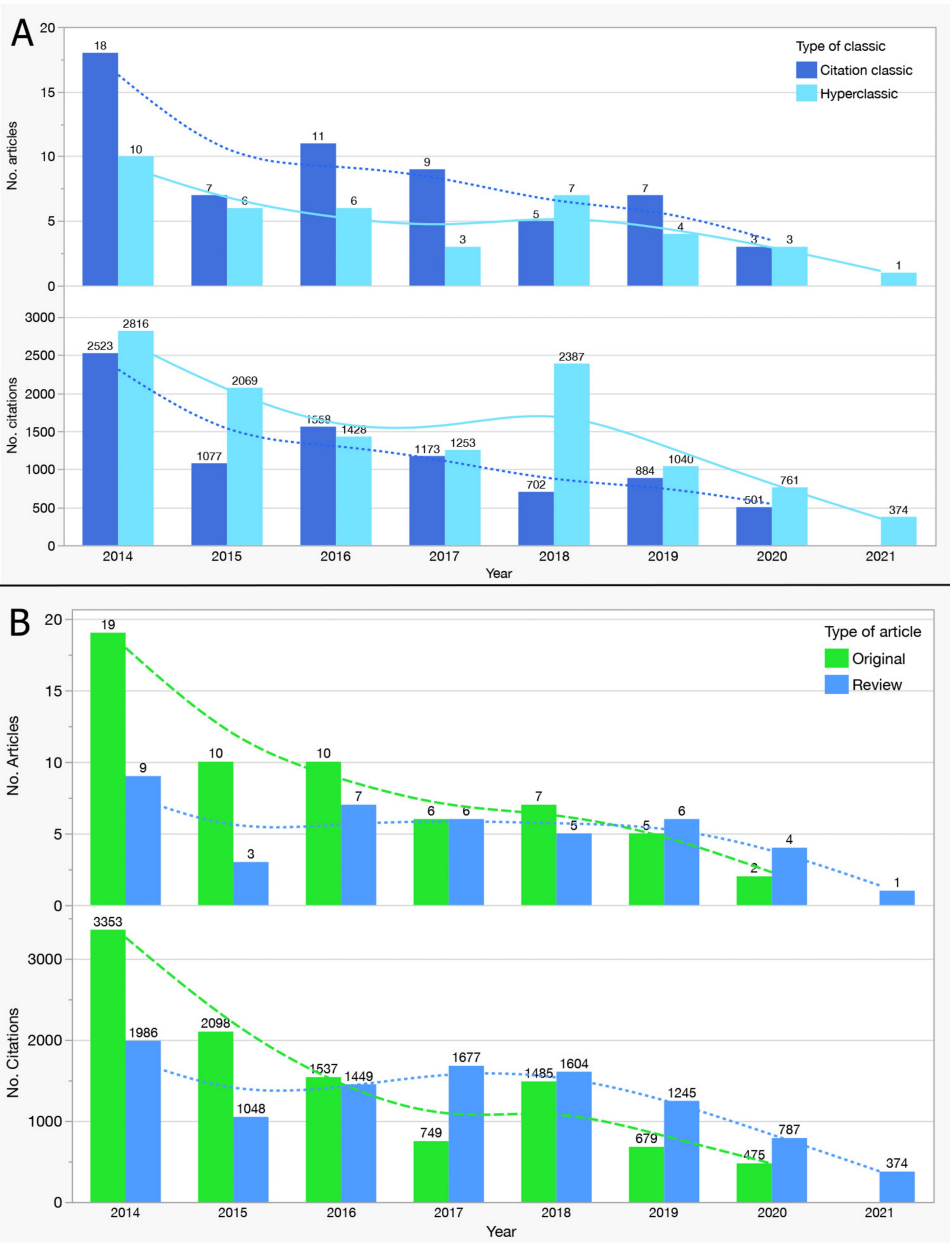
Yearly Distribution of Citation Classes and Article Types (Top 100 miRNA Articles in AD/PD, 2014–2021). (A) Annual counts of Citation Classics (CC; ≥100 citations) and Hyperclassics (HC; ≥250 citations) among the top 100 most-cited miRNA papers on cognition in AD/PD (upper panel). The lower panel shows total citations accrued by those papers per publication year. Smoothed lines depict temporal trends. (B) Annual counts of Original and Review articles (upper panel) and their year-grouped citations (lower panel), with smoothed trend lines. Axes: x-axis = publication year; y-axes = number of articles (upper panels) and total citations (lower panels). Colors correspond to in-figure legends. Abbreviations: AD, Alzheimer’s disease; PD, Parkinson’s disease; miRNA, microRNA; CC, Citation Classic (≥100 citations); HC, Hyperclassic (≥250 citations).

Authorship patterns are shown in Table 4. Nearly all papers were co-authored (99%; 1/100 sole-authored), and the distribution by class was not different (χ², p=0.218). Hyperclassics were more often reviews, whereas Classics were predominantly original articles (χ², p=0.002). The yearly distribution of Classics and Hyperclassics is shown in Figure 3.

**Table 4:** Categorical Characteristics by Citation Class. Values are n (%). Percentages for Hyperclassic and Classic are column percentages within each citation class; totals use N=100. Group comparisons used the chi-square (χ²) test. Two-tailed; p<0.05 considered significant.

Variable	Category	All articles (N=100)	Hyperclassic (N=40)	Classic (N=60)	p-value
Co-authorship	Co-authored	99 (99.0)	39 (97.5)	60 (100.0)	0.218
	Sole-authored	1 (1.0)	1 (2.5)	0 (0.0)	
Type of article	Original	59 (59.0)	16 (40.0)	43 (71.7)	0.002
	Review	41 (41.0)	24 (60.0)	17 (28.3)	

Citations by Journals and Journal-Level Metrics

The 100 articles appeared across 69 journals. Molecular Neurobiology contributed the highest total citations (1,232, 6.0%), followed by Progress in Neurobiology (1,093) and PLoS ONE (732) (Table 5). Journals with the highest average citations/year included Stem Cell Research & Therapy (83.0) and Molecular Therapy - Nucleic Acids (75.0). Median journal indicators were JIF 5.1 and CiteScore 10.0, consistent with publication in mid-to-high impact venues (Table 6).

**Table 5:** Top Journals Publishing Research on miRNAs and Cognitive Decline in AD and PD. Lists the leading journals based on the total number of citations, highlighting those that are most influential in disseminating research findings on miRNAs and neurodegenerative diseases. Abbreviations: AD, Alzheimer’s disease; PD, Parkinson’s disease; miRNA, microRNA.

Rank	Journal	Articles			Citations		
		Total	Original	Review	Total	%	Average/year
1	Molecular Neurobiology	7	3	4	1232	6.0%	21.9
2	Progress in Neurobiology	5	0	5	1093	5.3%	31.2
3	PLoS ONE	3	3	0	732	3.6%	23.7
4	Journal of Alzheimer's Disease	4	4	0	691	3.4%	16.8
5	Molecular Therapy Nucleic Acids	1	0	1	601	2.9%	75.0
6	Stem Cell Research and Therapy	1	0	1	578	2.8%	83.0
7	Molecular Psychiatry	3	3	0	566	2.8%	20.7
8	Biological Psychiatry	2	1	1	532	2.6%	58.5
9	Oncotarget	2	2	0	524	2.6%	27.5
10	Frontiers in Bioscience - Scholar	1	0	1	495	2.4%	
11	Frontiers in Neuroscience	2	1	1	473	2.3%	35.5
12	Frontiers in Genetics	2	0	2	463	2.3%	26.0
13	Neurobiology of Aging	3	3	0	457	2.2%	14.0
14	BMC Medicine	1	0	1	440	2.1%	55.0
15	Neuroscience and Biobehavioral Reviews	1	0	1	402	2.0%	40.0
16	International Journal of Molecular Sciences	2	0	2	398	1.9%	22.0
17	Theranostics	1	1	0	389	1.9%	56.0
18	Frontiers in Cellular Neuroscience	3	2	1	379	1.8%	11.3
19	Cell Death and Differentiation	1	0	1	362	1.8%	36.0
20	Physiological Reviews	1	1	0	361	1.8%	33.0
21	Expert Review of Molecular Diagnostics	1	0	1	331	1.6%	47.0
22	Human Molecular Genetics	2	2	0	299	1.5%	17.0
23	Journal of the Neurological Sciences	2	1	1	296	1.4%	14.5
24	Nature	1	1	0	294	1.4%	59.0
25	Cells	1	0	1	290	1.4%	48.0
26	Nature Reviews Neurology	1	0	1	289	1.4%	32.0
27	Bioinformatics	1	1	0	264	1.3%	38.0
28	Genes	1	1	0	255	1.2%	28.0
29	Alzheimer's and Dementia	1	0	1	247	1.2%	22.0
30	Scientific Report	1	1	0	247	1.2%	35.0
31	Frontiers in Aging Neuroscience	2	2	0	246	1.2%	15.5
32	EMBO Journal	1	1	0	245	1.2%	22.0
33	Journal of Translational Medicine	1	0	1	240	1.2%	48.0
34	Translational Neurodegeneration	2	2	0	233	1.1%	16.0
35	Frontiers in Molecular Neuroscience	2	1	1	232	1.1%	10.5
36	Nature Reviews Drug Discovery	1	0	1	230	1.1%	33.0
37	Frontiers in Immunology	1	0	1	227	1.1%	45.0
38	Biochimica et Biophysica Acta - Molecular Basis of Disease	1	0	1	224	1.1%	25.0
39	Nature Neuroscience	1	1	0	224	1.1%	37.0
40	Clinical Pharmacology and Therapeutics	1	1	0	213	1.0%	24.0
41	Neuron	1	0	1	212	1.0%	27.0
42	Journal of Neuroscience	1	1	0	206	1.0%	19.0
43	Biomedical and Environmental Sciences	1	1	0	185	0.9%	26.0
44	Nature Genetics	1	1	0	181	0.9%	36.0
45	Brain Pathology	1	1	0	172	0.8%	19.0
46	Molecular Brain	1	0	1	172	0.8%	29.0
47	Molecular Medicine Reports	1	1	0	166	0.8%	15.0
48	Disease Markers	1	1	0	165	0.8%	17.0
49	Journal of Clinical Investigation	1	1	0	162	0.8%	16.0
50	Molecules	1	0	1	160	0.8%	15.0
51	Journal of Biological Chemistry	1	1	0	158	0.8%	14.0
52	Neuroscience Letters	1	1	0	154	0.7%	19.0
53	Open Biology	1	0	1	153	0.7%	19.0
54	Journal of Biomedical Science	1	0	1	152	0.7%	30.0
55	Journal of Controlled Release	1	1	0	140	0.7%	16.0
56	FEBS Letters	1	1	0	139	0.7%	14.0
57	Brain Research	1	1	0	138	0.7%	15.0
58	Biomarker Insights	1	1	0	135	0.7%	14.0
59	Parkinsonism and Related Disorders	1	1	0	133	0.6%	15.0
60	Cellular and Molecular Life Sciences	1	0	1	130	0.6%	12.0
61	Acta neuropathologica communications	1	1	0	125	0.6%	16.0
62	Biomedical Journal	1	0	1	117	0.6%	17.0
63	Proceedings of the National Academy of Sciences of the United States of America	1	1	0	117	0.6%	12.0
64	Philosophical Transactions of the Royal Society B: Biological Sciences	1	0	1	116	0.6%	11.0
65	RNA	1	1	0	116	0.6%	11.0
66	Neuroscience Bulletin	1	1	0	115	0.6%	19.0
67	EMBO Molecular Medicine	1	1	0	113	0.5%	13.0
68	Journal of Neuroscience Research	1	1	0	111	0.5%	10.0
69	Current Opinion in Neurobiology	1	0	1	109	0.5%	14.0

**Table 6:** Correlation of Journal Impact Metrics with Citation Performance. This table shows the correlation coefficients between various impact metrics (Impact Factor, CiteScore, SJR, SNIP) and the citation performance of articles on miRNAs related to cognitive decline. Abbreviations: AD, Alzheimer’s disease; PD, Parkinson’s disease; miRNA, microRNA, SJR: SCImago Journal Rank, SNIP: Source Normalized Impact per Paper, IF: Impact Factor.

	Median	Q1	Q3	IQR
IF	5.1	3.55	9.4	5.85
CiteScore	10	6.9	17.7	10.8
SJR	1.798	1.136	2.912	1.776
SNIP	1.294	0.9	1.963	1.063
Quartile	Q1	Q3	Q4	Q1-Q2

**Figure 4:**
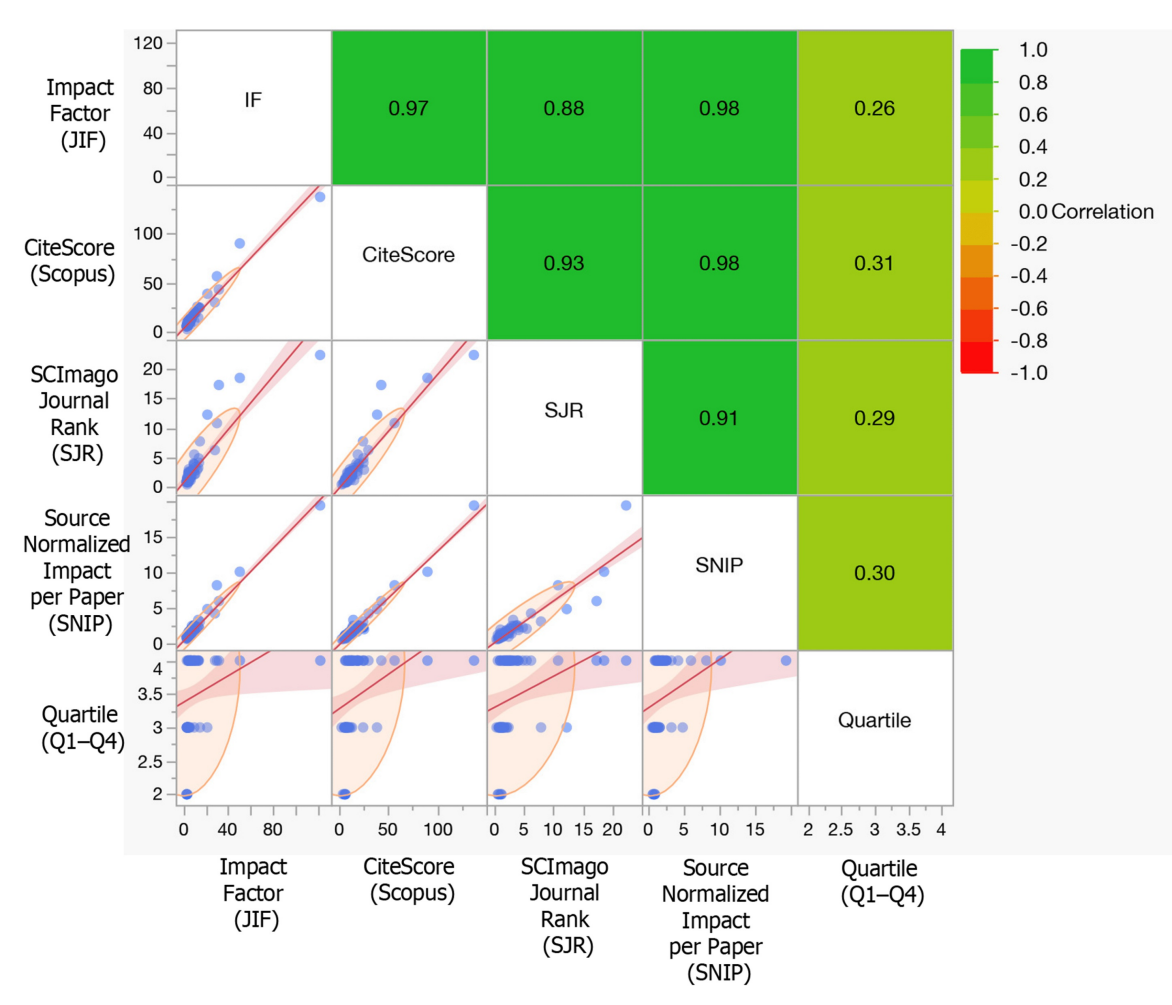
Correlation Matrix and Pairwise Relationships Among Journal Metrics. The matrix summarizes relationships among Impact Factor (JIF), CiteScore (Scopus), SCImago Journal Rank (SJR), Source Normalized Impact per Paper (SNIP), and Quartile. Upper-triangle cells display Pearson correlation coefficients (r) with a diverging color scale (−1 to +1; see side legend). Lower-triangle panels show pairwise scatterplots with fitted trend and confidence band; orange ellipses indicate the concentration of points. Axes are labeled by column metric (x-axis) and row metric (y-axis). Quartile is ordinal (Q1 = 1, Q4 = 4; lower values indicate higher rank). Overall, the numerical indices (JIF, CiteScore, SJR, SNIP) exhibit strong positive associations, while correlations with Quartile are modest.

Inter-metric correlations were strong and positive among JIF, CiteScore, SJR, and SNIP (all p<0.001). Figure 4 displays a correlogram (upper triangle) with pairwise scatterplots (lower triangle). Axes are explicitly labeled; x-axis = column metric and y-axis = row metric. Quartile is coded Q1=1 (highest) to Q4=4.

Publications Grouped by Institutions

Harvard Medical School led total citations (four articles; 683 citations; 3.3% of total), followed by Texas Tech University Health Sciences Center (638) and Hallym University-Chuncheon Sacred Heart Hospital (601) (Table 7). Notably, Sorbonne University produced the highest average citations/year (94.0) with a single, highly influential article.

**Table 7:** Leading Institutions in microRNA (miRNA) Research on Cognitive Decline. This table ranks institutions based on the total number of citations and publications, showcasing major contributors to the field of miRNA research in Alzheimer's and Parkinson's diseases.

Rank	Institution	Articles	Citations		
			N	%	Average per year
1	Harvard Medical School	4	683	3.3%	24.0
2	Texas Tech University Health Sciences Center	3	638	3.1%	29.7
3	Hallym University-Chuncheon Sacred Heart Hospital	1	601	2.9%	75.0
4	Shiraz University of Medical Sciences	1	578	2.8%	83.0
5	Central South University	2	512	2.5%	38.5
6	Rutgers University	1	495	2.4%	45.0
7	University of Belgrade	1	440	2.1%	55.0
8	Zhejiang University	1	412	2.0%	41.0
9	University of Lethbridge	1	402	2.0%	40.0
10	Sorbonne University	1	374	1.8%	94.0
11	Qingdao University	2	370	1.8%	17.0
12	University of Leicester	1	362	1.8%	36.0
13	The Weizmann Institute	1	361	1.8%	33.0
14	Capital Medical University	2	351	1.7%	20.5
15	Kansas University Medical Center	1	348	1.7%	50.0
16	University of Southern Maine	1	340	1.7%	31.0
17	University of Florida	1	331	1.6%	47.0
18	University of Illinois at Chicago	1	308	1.5%	31.0
19	The University of Melbourne	1	301	1.5%	30.0
20	Nanjing University	2	298	1.5%	16.0
21	University of California	1	294	1.4%	59.0
22	University of Oxford	1	289	1.4%	32.0
23	Maastricht University	1	284	1.4%	28.0
24	Montréal Neurological Institute	1	277	1.3%	46.0
25	Indiana University School of Medicine	2	266	1.3%	16.0
26	University of Louisville	1	265	1.3%	24.0
27	China University of Mining and Technology	1	264	1.3%	38.0
28	Louisiana State University Health Science Center	1	255	1.2%	28.0
29	Loughborough University	1	249	1.2%	42.0
30	Columbia University Medical Center	1	247	1.2%	35.0
31	Nordic Bioscience Biomarkers and Research	1	247	1.2%	22.0
32	German Center for Neurodegenerative Diseases	1	245	1.2%	22.0
33	Translational Genomics Research Institute	1	240	1.2%	22.0
34	Urmia University of Medical Sciences	1	240	1.2%	48.0
35	University Medical Center Utrecht	1	230	1.1%	26.0
36	Voyager Therapeutics	1	230	1.1%	33.0
37	Washington University School of Medicine	1	224	1.1%	37.0
38	University of Cambridge	1	217	1.1%	24.0
39	Queen's Medical Research Institute	1	213	1.0%	24.0
40	Tohoku University	1	213	1.0%	19.0
41	Washington University in St. Louis	1	212	1.0%	27.0
42	Radboud University Medical Centre	1	204	1.0%	19.0
43	RWTH Aachen University	1	199	1.0%	22.0
44	University Hospital Hamburg-Eppendorf	1	184	0.9%	18.0
45	La Trobe University	1	181	0.9%	20.0
46	University of Pennsylvania	1	181	0.9%	36.0
47	Université Laval	1	175	0.9%	18.0
48	University of Milan	1	175	0.9%	16.0
49	Southern Medical University	1	172	0.8%	19.0
50	University of Gothenburg	1	172	0.8%	29.0
51	Medicine and Life Science Maastricht University	1	168	0.8%	34.0
52	Columbia University	1	162	0.8%	16.0
53	Shanghai Jiao Tong University	1	162	0.8%	20.0
54	University of Trento	1	160	0.8%	15.0
55	Huazhong University of Science and Technology	1	158	0.8%	23.0
56	University of Queensland	1	157	0.8%	14.0
57	Nanjing Medical University	1	154	0.7%	19.0
58	Medical University of Gdansk	1	153	0.7%	19.0
59	Academia Sinica	1	152	0.7%	30.0
60	University of Beira Interior	1	140	0.7%	16.0
61	IRCCS Hospital San Camillo	1	139	0.7%	13.0
62	Rutgers - Robert Wood Johnson Medical School	1	139	0.7%	14.0
63	West Virginia University	1	138	0.7%	15.0
64	Meiji Pharmaceutical University	1	135	0.7%	14.0
65	Tongji University School of Medicine	1	134	0.7%	22.0
66	University of Helsinki	1	130	0.6%	12.0
67	University of Perugia	1	128	0.6%	12.0
68	Copenhagen University Hospital	1	126	0.6%	14.0
69	Universidad Autónoma de Madrid	1	126	0.6%	11.0
70	Advanced Genomic Technology	1	125	0.6%	11.0
71	Boston University School of Medicine	1	125	0.6%	14.0
72	Niigata University	1	125	0.6%	16.0
73	Radboud University Medical Center	1	122	0.6%	14.0
74	Union College	1	121	0.6%	17.0
75	University of Toronto	1	121	0.6%	17.0
76	University Hospital Marqués de Valdecilla	1	120	0.6%	15.0
77	Foundation for Biomedical Research and Innovation	1	117	0.6%	12.0
78	University of the Pacific	1	117	0.6%	17.0
79	CSIR-Institute of Genomics and Integrative Biology	1	116	0.6%	11.0
80	The Philipps University of Marburg	1	116	0.6%	11.0
81	Huazhong University of Science and Technology	1	115	0.6%	19.0
82	Academy of Athens	1	114	0.6%	10.0
83	August Pi i Sunyer Biomedical Research Institute	1	114	0.6%	14.0
84	VIB Center for the Biology of Disease	1	113	0.5%	13.0
85	Polish Academy of Sciences	1	112	0.5%	14.0
86	Centro de Investigacion sobre Enfermedades Neurodegenerativas	1	111	0.5%	10.0
87	Gachon University	1	109	0.5%	12.0
88	Johns Hopkins University	1	109	0.5%	14.0
89	Aix-Marseille Université	1	107	0.5%	10.0
90	Health Sciences Research Institute Germans Trias i Pujol	1	107	0.5%	18.0

Publications Grouped by Country

**Figure 5:**
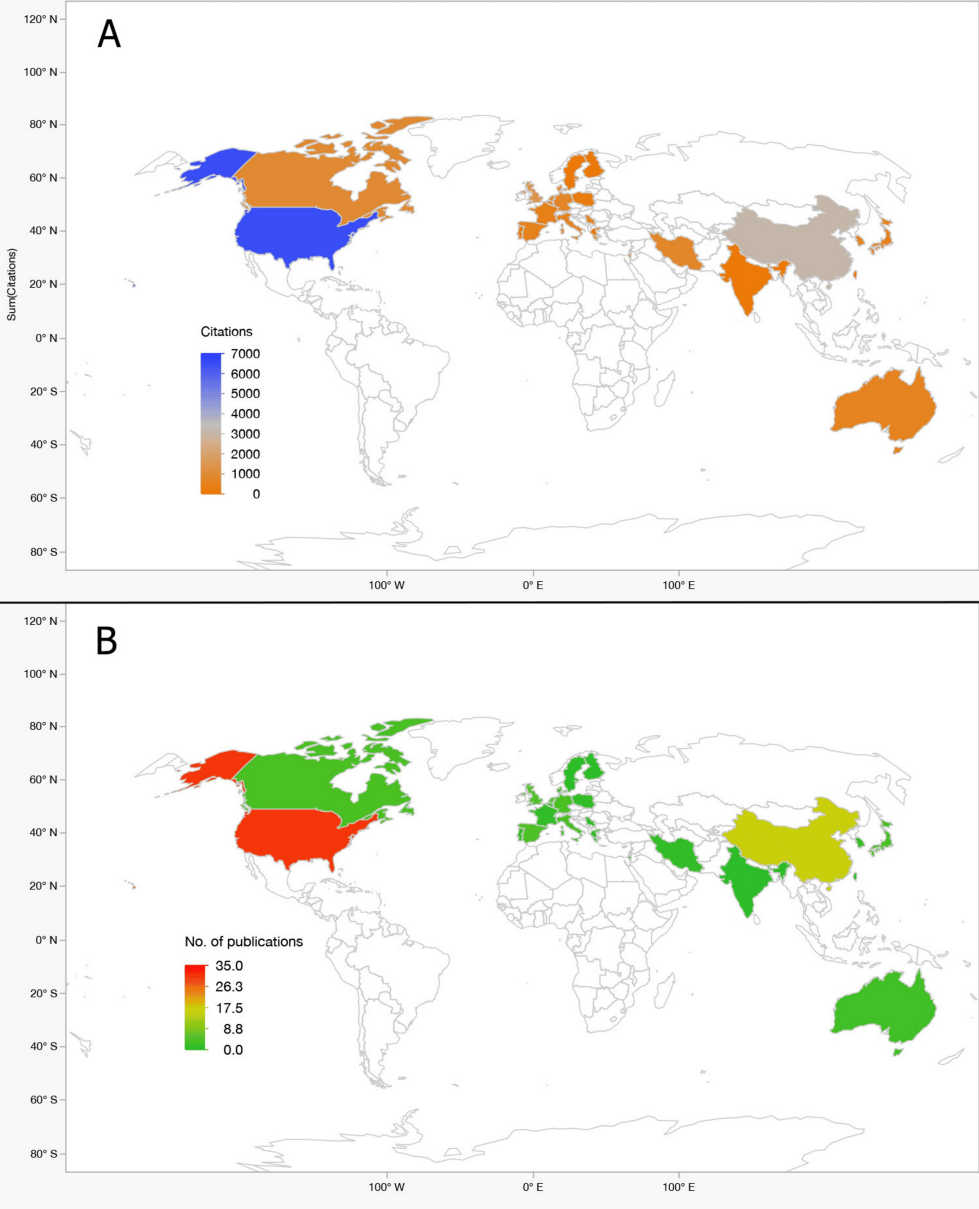
Global Distribution of Citations and Publications (Top 100 miRNA Articles in AD/PD, 2014–2024). World maps showing country contributions among the top 100 most-cited miRNA papers related to cognition in Alzheimer’s disease (AD) and Parkinson’s disease (PD). (A) Choropleth of total citations per country (at data-extraction), highlighting leading contributors. (B) Choropleth of number of publications per country in the same cohort. Color bars indicate relative magnitude within each panel; countries with no records are unshaded. Abbreviations: AD, Alzheimer’s disease; PD, Parkinson’s disease; miRNA, microRNA.

As shown in Table 8, the United States dominated output and impact (31 articles; 6,593 citations; 32.1% of total), followed by China (16 articles; 3,102 citations). Other major contributors included the United Kingdom, Germany, and Canada. Figure 5 visualizes both citations (panel A) and publications (panel B) geographically.

**Table 8:** Global Distribution of Research Output on microRNAs (miRNAs) and Cognitive Decline. The table summarizes the number of publications and citations from different countries, highlighting the global contributions to miRNA research in neurodegenerative diseases.

Ranking	Country	Articles		Citations		
			Number		%	Average per year
1	United States	31	6593		32.1%	27.1
2	China	16	3102		15.1%	24.1
3	United Kingdom	5	1330		6.5%	31.6
4	Netherlands	5	1008		4.9%	24.2
5	Canada	4	975		4.7%	30.3
6	Iran	2	818		4.0%	65.5
7	Germany	4	744		3.6%	18.3
8	South Korea	2	710		3.5%	43.5
9	Australia	3	639		3.1%	21.3
10	Italy	4	602		2.9%	14.0
11	Japan	4	590		2.9%	15.3
12	Spain	5	578		2.8%	13.6
13	France	2	481		2.3%	52.0
14	Serbia	1	440		2.1%	55.0
15	Denmark	2	373		1.8%	18.0
16	Israel	1	361		1.8%	33.0
17	Poland	2	265		1.3%	16.5
18	Sweden	1	172		0.8%	29.0
19	Taiwan	1	152		0.7%	30.0
20	Portugal	1	140		0.7%	16.0
21	Finland	1	130		0.6%	12.0
22	India	1	116		0.6%	11.0
23	Greece	1	114xc		0.6%	10.0
24	Belgium	1	113		0.5%	13.0

Science Mapping of Cognitive-Related miRNAs in AD and PD

**Figure 6:**
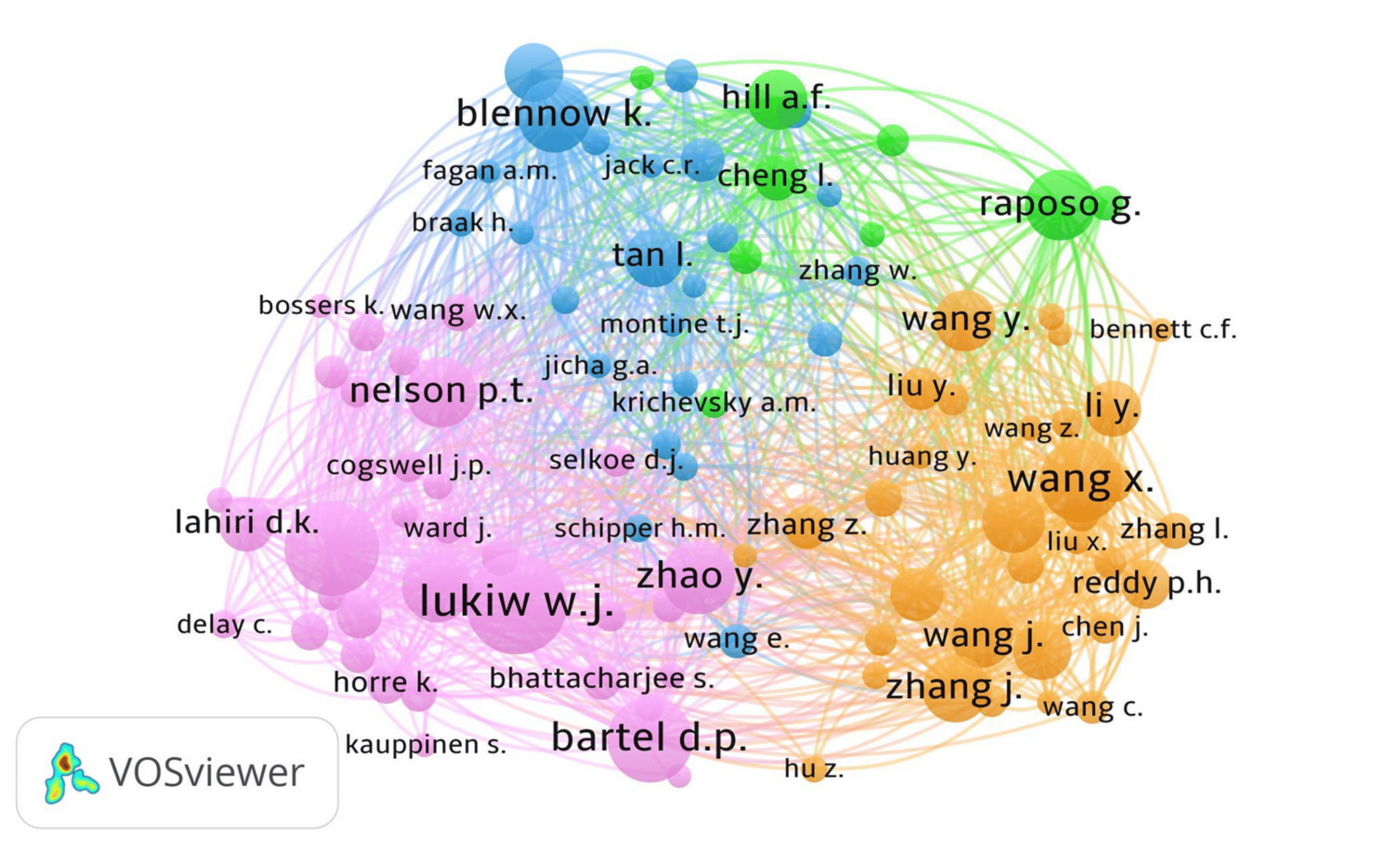
Author Co-citation Network (Top 100 miRNA Articles in AD/PD). Network map of author co-citations among the top 100 most-cited miRNA papers related to cognition in Alzheimer’s disease (AD) and Parkinson’s disease (PD). Each node (circle) is an author; node size is proportional to the author’s total co-citation link strength (overall frequency/weight of being cited together with others). Node color denotes the cluster/community identified by VOSviewer. Edge thickness represents the co-citation link strength between two authors; node proximity reflects relatedness. Labels are shown for the most central nodes. Only authors meeting the minimum inclusion threshold are displayed. Abbreviation: miRNA, microRNA.

Intellectual structure (author co-citation): The co-citation analysis identified 22,456 authors; applying a minimum 25-citation threshold yielded 67 influential authors grouped into four clusters. Prominent nodes included Bartel D.P., Wang X., Raposo G., and Blennow K. (Figure 6).

Network conventions: Node size = total link strength/occurrence, node color = cluster/community, edge thickness = link strength; spatial proximity reflects relatedness.

Key Concepts and Their Relationships (Keyword Co-occurrence)

**Figure 7:**
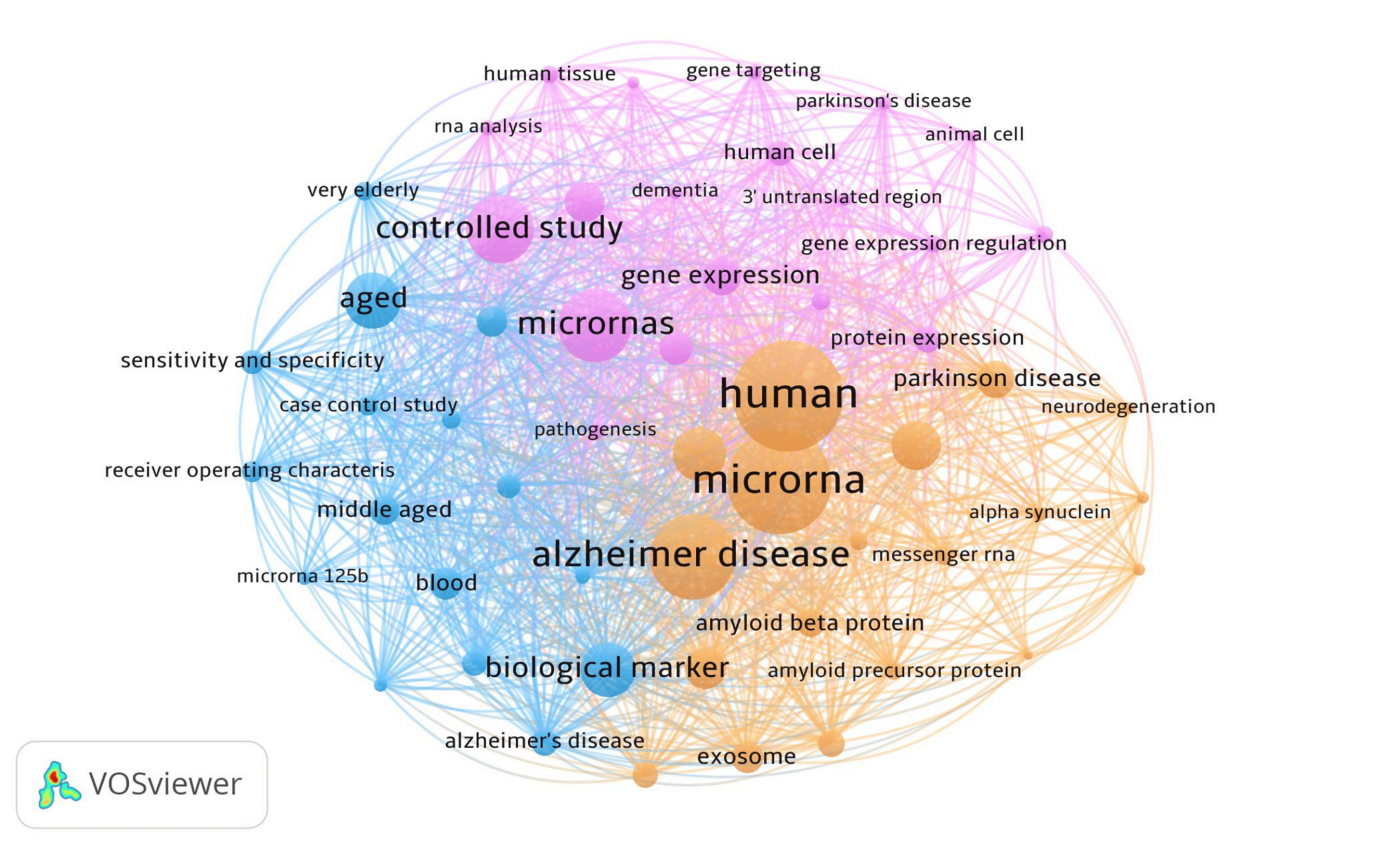
Keyword Co-occurrence Network (Research Themes in the Top-100 miRNA Articles in AD/PD). Network map of author keywords/Keywords Plus showing co-occurrence patterns in the top-100 most-cited miRNA papers related to cognition in Alzheimer’s disease (AD) and Parkinson’s disease (PD). Each node is a keyword; node size is proportional to its occurrence frequency/total link strength in the dataset. Node color denotes the cluster/community (topic) detected by VOSviewer. Edge thickness represents the co-occurrence link strength between two keywords; spatial proximity reflects relatedness. Labels are shown for high-frequency terms; only terms meeting the inclusion threshold are displayed. Abbreviations: AD, Alzheimer’s disease; PD, Parkinson’s disease; miRNA, microRNA.

From 1,904 keywords, a threshold of ≥10 occurrences yielded 54 terms forming three clusters (Figure 7). Cluster 1 encompassed disease pathogenesis (e.g., alpha-synuclein, amyloid-β, neurodegeneration); Cluster 2 grouped miRNA and gene-regulation terms with study-design descriptors; Cluster 3 included translational terms such as biological marker and aged.

Nature of Collaboration (Co-authorship)

**Figure 8:**
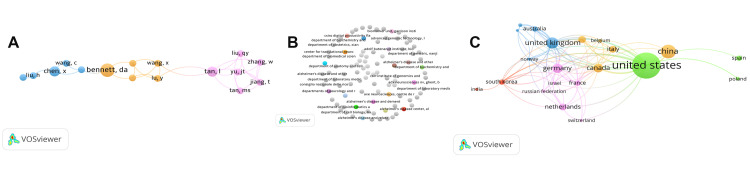
Co-authorship Networks by Author, Organization, and Country (Top 100 miRNA Articles in AD/PD). Network visualizations of co-authorship within the top 100 most-cited miRNA papers related to cognition in Alzheimer’s disease (AD) and Parkinson’s disease (PD), produced in VOSviewer. (A) Authors. Each node is an author. Node size ∝ number of documents authored in the set (weighted by fractional counting). Node color = cluster/community. Edge thickness ∝ co-authorship link strength (number/weight of joint publications). (B) Organizations. Nodes represent institutions; sizing, coloring, and edge meanings as above (output and collaboration strength at the organization level). (C) Countries. Nodes represent countries; node size - national output (documents in the set), edge thickness - international collaboration link strength; colors indicate clusters of closely collaborating countries. Spatial proximity reflects relatedness in each map. Only entities meeting inclusion thresholds are displayed. Abbreviations: AD, Alzheimer’s disease; PD, Parkinson’s disease; miRNA, microRNA.

The co-authorship network included 595 authors, with 37 meeting the threshold of two or more documents (Figure 8A). Organization- and country-level maps revealed broad, multi-center collaboration (418 organizations; 35 countries), with hubs in the United States, China, and Europe (Figure 8B-8C).

Temporal Research Themes

**Figure 9:**
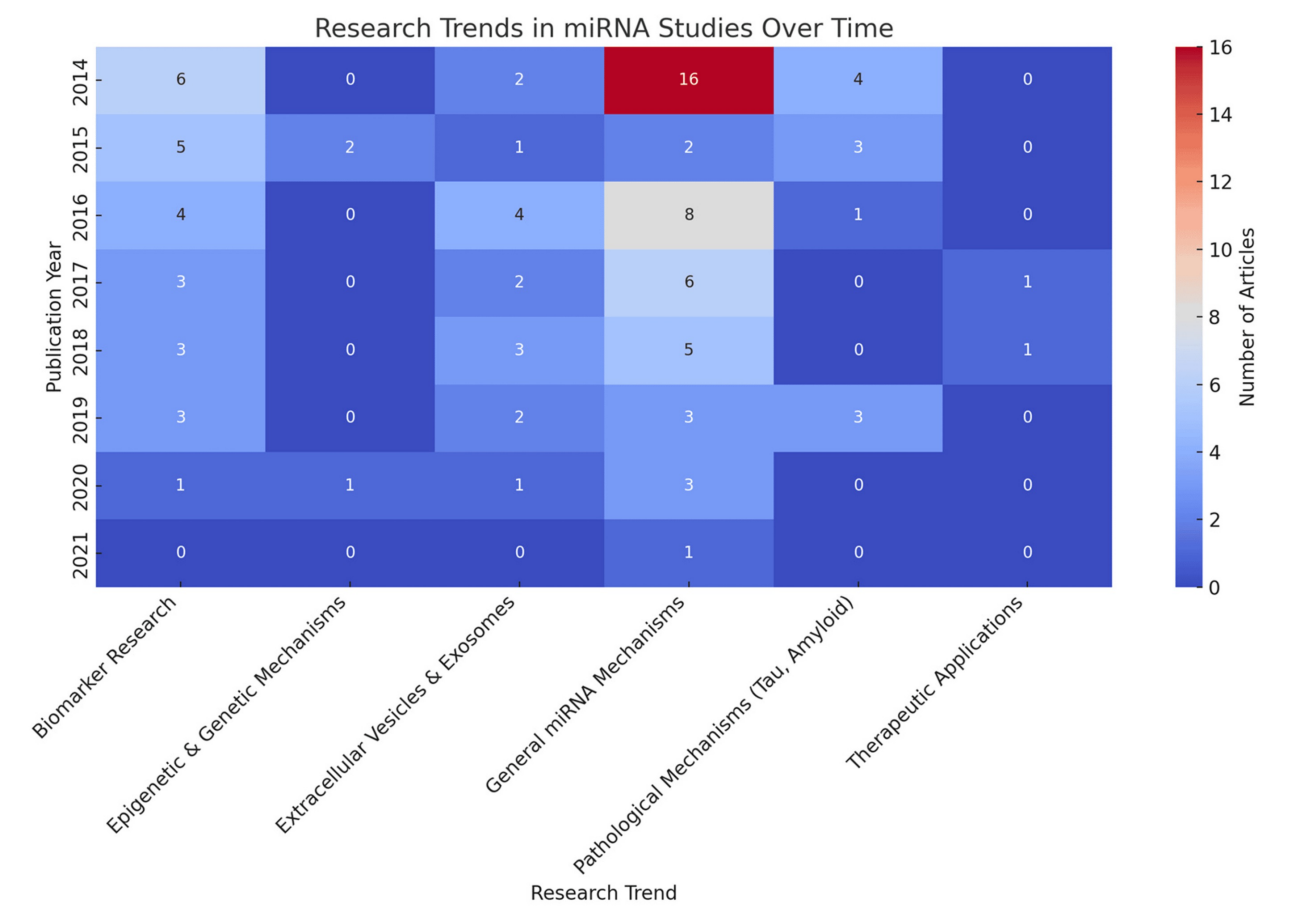
Temporal Heatmap of Research Themes in microRNA (miRNA) Articles on Cognitive Decline (2014–2021). Heatmap showing annual counts of articles across research themes within the top 100 most-cited miRNA papers on cognition in Alzheimer’s disease (AD) and Parkinson’s disease (PD). The y-axis lists publication years (2014→2021) and the x-axis lists themes: Biomarker Research; Epigenetic & Genetic Mechanisms; Extracellular Vesicles & Exosomes; General miRNA Mechanisms; Pathological Mechanisms (Tau, Amyloid); Therapeutic Applications. Cell annotations indicate number of articles per year–theme. The color scale (blue→red) reflects intensity, with warmer colors indicating higher counts. The map highlights shifts from early emphasis on pathological/general mechanisms toward biomarkers, exosomes, and therapeutic applications in later years. This view highlights emergence (first active year) and peaks, summarized in Table 9.

The heatmap (Figure 9) shows a progression from general/mechanistic miRNA studies early in the period to later emphasis on biomarkers, extracellular vesicles and exosomes, and therapeutic applications, indicating maturation toward translational research.

**Table 9:** Emergent miRNA themes—volume, timing, and citation impact (top 100 corpus, 2014–2021). Note: Dashes indicate descriptive counts without pooled citation reporting in the source summary; all values derive from the curated top 100 dataset used for Figure 9.

Theme	Articles (n)	Emergence / Peak year	Total citations	Avg citations per article
Biomarker Research	24	2014 / 2014	5421	226
Extracellular Vesicles & Exosomes	14	2016 / 2016	2308	165
Pathological Mechanisms (Tau, Amyloid)	10	2014 / 2014	—	—
Epigenetic & Genetic Mechanisms	6	2015 / —	—	—
Therapeutic Applications	5	2017 / 2019	834	167
General miRNA Mechanisms	37	2014 / 2014	—	—

To highlight theme emergence, we summarized for each category the first year with visible activity and its subsequent peak. Biomarker Research emerged and peaked in 2014 (six papers; cumulative 24 across the window). Extracellular Vesicles & Exosomes emerged in 2016 (peak four papers) with sustained activity in 2018-2020 (three per year; total 14). Therapeutic Applications appeared in 2017 and peaked in 2019 (three papers; total five). Pathological Mechanisms (Tau/Amyloid) peaked early in 2014 (four papers; total 10) and tapered thereafter. Epigenetic & Genetic Mechanisms began in 2015 and remained low but steady (total six). General miRNA Mechanisms showed an early exploratory peak in 2014 (16 papers) before narrowing toward translational threads by 2018 (total 37) (summary in Table 9).

These trajectories are also compatible with vascular cognitive impairment, where inflammatory and endothelial miRNA signals, often captured in blood or EVs, are expected to rise early after ischemic injury and evolve with small-vessel disease burden.

Discussion

Overview and Clinical Relevance

This bibliometric-scoping review distilled the top 100 most-cited studies on miRNAs associated with cognitive decline in AD and PD from an initial pool of 7,722 records. Focusing on influence (citations) rather than evidence tier, the set captures how the field has shaped clinical thinking about early detection, risk stratification, and treatment monitoring. Because miRNAs circulate in accessible biofluids and regulate gene networks central to neurodegeneration, they remain strong biomarker candidates for cognitive impairment and its progression.

Performance Trends

Publication activity averaged 12.5 articles/year across 2014-2021, peaking in 2014 (28 papers; 5,339 citations) (Figure 2). Topics prominent around the peak included extracellular vesicles and exosomes, particularly neuronal-derived extracellular vesicles (NDEVs), which mirror CNS processes and carry disease-relevant cargo [129,130]. Released by neurons, astrocytes, and microglia, NDEVs mediate intercellular communication and may reflect neurodegenerative or neuroprotective states [131-134]. Cargo such as Aβ, p-tau, and neurofilament supports their diagnostic and monitoring potential in AD [135]. The absence of 2022-2024 items in the top-100 is expected given citation accrual dynamics and a shift toward diversified lines of inquiry.

Citation Classes and Interpretability

Hyperclassics (≥250 citations; N=40) accrued more total and annual citations than Classics (≥100 citations; N=60), with no differences in years active or author counts (Table 3; Figure 3). Hyperclassics were more often reviews, whereas Classics were more frequently original studies. This pattern is common in emerging translational fields: integrative reviews guide programs of research and attract sustained citations, while original studies provide the empirical substrate. These findings highlight influence, not necessarily higher evidentiary certainty, and should motivate prospective validation of key claims.

Journals and Journal-Level Metrics

The corpus spanned 69 journals; Molecular Neurobiology, Progress in Neurobiology, and PLoS ONE led total citations (Table 5). Median indicators (JIF 5.1; CiteScore 10.0) reflect publication in mid-to-high impact venues (Table 6). Inter-metric correlations among JIF, CiteScore, SJR, and SNIP were strong (all p<0.001), whereas quartile modestly tracked other indicators (Figure 4). Thus, within this niche, topical fit and audience appear to matter as much as rank surrogates.

Institutional and Geographic Concentration

Institutions with the largest influence included Harvard Medical School, Texas Tech University Health Sciences Center, and Hallym University-Chuncheon Sacred Heart Hospital (Table 7). The United States dominated both output and citations, followed by China and key European contributors (Table 8, Figure 5). High average-citations/year from countries with fewer publications (e.g., Iran, Serbia) illustrate that quality and specificity can outweigh volume. Broadening participation - especially to under-represented regions - will be essential for biomarker validation across diverse genetic and environmental backgrounds. Regional collaboration showed dense North America-Europe links with increasingly strong ties involving Asia (notably China). Cross-continental bridges were common among high-output hubs, a pattern that supports multi-site validation and technology transfer.

Science Mapping: Structure, Concepts, and Collaboration

Co-citation mapping (Figure 6) resolved four author clusters anchored by leaders in RNA biology, neuropathology, and biomarker development (e.g., Bartel D.P., Wang X., Raposo G., Blennow K.). Keyword co-occurrence (Figure 7) organized three themes: (1) pathogenesis (e.g., alpha-synuclein, amyloid-β, exosome), (2) gene-regulatory and study-design terms, and (3) translational biomarker concepts (e.g., biological marker, aged). Co-authorship networks (Figure 8A-8C) showed multi-center, international collaboration, with hubs in the United States, China, and Europe and numerous cross-regional links - an encouraging substrate for multi-site validation [136-138].

Temporal Research Themes

The heatmap (Figure 9, Table 9) indicates a shift from general/mechanistic miRNA work in early years to later emphasis on biomarkers, extracellular vesicles & exosomes, and therapeutic applications. This trajectory is consistent with maturation toward clinical translation. Highly cited studies disproportionately emphasize blood-based miRNA panels and extracellular-vesicle cargo, aligning with clinical priorities for minimally invasive early detection and longitudinal monitoring in cognitive decline. These trajectories align with translation: early biomarker activity concentrated on blood-based miRNA panels, whereas the later rise of EV/exosome work reflects interest in vesicle cargo as a minimally invasive readout of CNS processes. The smaller but growing therapeutic thread coincides with delivery-platform advances and target prioritization.

Relevance to Post-stroke Cognitive Impairment and Vascular Dementia

The dominant patterns in this corpus - blood-based and EV-associated miRNA readouts; emphasis on inflammatory, endothelial, and neurovascular signaling; and movement toward translational panels - map naturally to PSCI and VaD. Canonical vascular and injury-responsive miRNAs (e.g., miR-126 for endothelial homeostasis, miR-210 for hypoxia-angiogenesis; miR-21/miR-155/miR-146a/miR-223 for inflammatory/immune pathways; miR-124 for neuronal injury) are detectable in plasma/serum and extracellular vesicles and could support early risk stratification after stroke, trajectory monitoring across acute-subacute-chronic phases, and subtype differentiation (small-vessel disease vs strategic infarct patterns). In practice, next-step studies should test combined models - miRNA panels plus vascular risk factors and imaging markers (e.g., white-matter hyperintensities, lacunes) - and evaluate clinical utility for predicting cognitive outcomes and guiding secondary prevention.

Recent Advances Bridging the Corpus and the Present

While our analytic window ends in 2021, several recent findings further the translational arc. Blood-based miRNA panels for pre-symptomatic AD have been reported [139]; miR-129-5p associates with AD neuropathology and cognitive decline, with machine-learning-assisted models improving discrimination [20]. In PD, miR-203a-3p shows promise for predicting dementia [140]. Convergent miRNA-regulated pathways in AD and PD suggest shared mechanisms [141], and interactions between miRNAs and membrane transporters may contribute to AD pathogenesis [142]. Therapeutically, curated miRNA target signatures are being advanced as candidates for intervention in both disorders [143]. Together, these observations reinforce the diagnostic and theranostic potential of miRNAs. Content and impact were AD-predominant, with PD studies comparatively fewer and more heterogeneous. Translational readiness appears greater in AD (earlier movement toward blood-based signatures and EV-derived markers), whereas PDbiomarker work - particularly for PD dementia - is emerging and under active development.

Risk of Bias and Limitations

Strengths include PRISMA-style reporting, dual-reviewer screening, and integration of performance metrics with network science. This citation-based sampling reflects influence rather than evidentiary strength. Limitations are inherent to citation-based sampling (influence ≠ evidence hierarchy), potential self-citation effects, language/database bias (English, Scopus), and heterogeneity in assays and normalization across studies. The review is descriptive and exploratory (no meta-analysis or meta-regression), so associations should be viewed as hypothesis-generating. Expanding to multi-database sources and incorporating prospective clinical validation will provide a more holistic view.

Implications and Future Directions

For clinicians, the convergence on circulating miRNAs/NDEVs underscores near-term opportunities in early detection, risk staging, and treatment monitoring. For researchers, priorities include standardized pre-analytics/analytics, transparent normalization, multi-ethnic cohorts, and prospective studies linking miRNA signatures to cognitive outcomes. For policy and funders, the geographic concentration of influence argues for capacity building and cross-regional consortia to accelerate equitable translation. Next steps should include multi-omics bibliometric mapping (integrating proteomics, metabolomics, and epigenomics) and AI-driven citation/network modeling to forecast emerging biomarker themes and prioritize validation targets. Harmonized pre-analytics, multi-ethnic prospective cohorts, and clinically anchored endpoints will be essential to move miRNA panels from promise to practice.

## Conclusions

This bibliometric-scoping review of the top-100 most-cited studies on miRNAs and cognitive decline in Alzheimer’s disease and Parkinson’s disease maps the intellectual, journal, and collaborative structure of the field. The corpus shows a clear trajectory from general/mechanistic work toward translational biomarker efforts - particularly circulating miRNAs and neuronal-derived extracellular vesicles - and confirms strong clustering among leading authors, institutions, and countries. Together, these patterns explain why certain themes and venues concentrate influence and how knowledge has propagated across groups.

To convert influence into clinical utility, the next phase should prioritize: standardized pre-analytic/analytic workflows and explicit normalization; multi-center, multi-ethnic prospective cohorts with harmonized cognitive outcomes; rigorous validation of panels that combine miRNAs with established markers; and integrative AI/machine-learning models linking miRNA signatures to prognosis and treatment response. Targeted expansion to other non-coding RNAs and systematic evaluation of extracellular vesicle cargo are natural extensions. With these steps, miRNAs can move from promising indicators to actionable biomarkers for early detection, risk stratification, and monitoring - ultimately improving patient-centered care in neurodegenerative disease.
